# Amino acid insertion in the Meq protein of Marek’s disease virus, an avian oncogenic herpesvirus, accelerates tumorigenesis

**DOI:** 10.1128/spectrum.03368-24

**Published:** 2025-07-17

**Authors:** Jumpei Sato, Shiro Murata, Yoshinosuke Motai, Shwe Yee Win, Hikari Seo, Shunsuke Yamagami, Naoya Maekawa, Tomohiro Okagawa, Satoru Konnai, Kazuhiko Ohashi

**Affiliations:** 1 Department of Disease Control, Faculty of Veterinary Medicine, Hokkaido University 12810, Sapporo, Japan; 2 Department of Advanced Pharmaceutics, Faculty of Veterinary Medicine, Hokkaido University 12810, Sapporo, Japan; 3 Institute for Vaccine Research and Development, Hokkaido University 12810, Sapporo, Japan; 4 International Affairs Office, Faculty of Veterinary Medicine, Hokkaido University 12810, Sapporo, Japan; Wayne State University, Detroit, Michigan, USA

**Keywords:** Marek's disease virus, Marek's disease, Meq, L-Meq, insertion, tumorigenesis, virulence, transactivation activity

## Abstract

Marek’s disease virus (MDV) causes lymphomas (Marek’s disease) in chickens. Despite vaccination, MDV field strains exhibit increased virulence, and sporadic outbreaks are still reported. An insertion/deletion in Meq has been identified in several MDV strains, and our previous study using recombinant MDV (rMDV) demonstrated that an insertion in Meq enhanced MDV virulence, whereas a deletion reduced its virulence. However, the mechanisms by which these indels in Meq alter MDV virulence remain elusive. We aimed to clarify the impact of the insertion in Meq on pathogenesis. We compared the effects of Meq and Meq with the insertion, termed L-Meq, on transcriptional regulation, the dynamics of transformed T cells and relevant T-cell subsets, and the patterns of gene expression in tumor lesions of infected chickens. Reporter assays revealed that insertion increased the transactivation activity on the infected-cell protein 4 promoter in the MDV genome and bcl-2/cd30 promoters in the host genome. rMDV encoding L-Meq (vRB-1B_L-Meq) exhibited higher mortality and tumor incidence than rMDV encoding Meq (vRB-1B_Meq), and vRB-1B_L-Meq infection increased the proportion of CD4^+^ T cells, which are targets for transformation by MDV, in the early post-infection phase, compared with vRB-1B_Meq. RNA-seq analysis revealed that similar genes were modulated in tumor lesions of chickens infected with vRB-1B_Meq or vRB-1B_L-Meq. These findings suggest that although the insertion in Meq does not alter the target genes for transcriptional regulation, it accelerates the process of tumorigenesis by enhancing the transactivation activity.

**IMPORTANCE:**

Marek’s disease, an avian lymphoproliferative disease, is caused by Marek’s disease virus (MDV). Meq, an MDV oncoprotein, regulates the expression of viral and host genes. *Meq* with an insertion, termed L-*Meq*, has been identified as a factor that enhances MDV virulence. However, the mechanisms by which the insertion in *Meq* alters MDV virulence remain unknown. Our study clarified that the insertion enhances the transactivation activity of Meq on the host promoters related to tumorigenesis. Notably, the transcriptomes of tumor lesions in chickens infected with recombinant MDV (rMDV) with L-*Meq* and those infected with rMDV encoding *Meq* without the insertion were similar; however, chickens infected with rMDV harboring L-*Meq* exhibited higher proportions of CD4^+^ T cells and regulatory T cells, which are targets for transformation by MDV, in the early post-infection phase, suggesting accelerated tumorigenesis. This study contributes to the current understanding of the mechanisms underlying MDV virulence.

## INTRODUCTION

Marek’s disease virus (MDV) is a widely prevalent poultry alphaherpesvirus that belongs to the *Orthoherpesviridae* family (subfamily: *Alphaherpesvirinae*, genus: *Mardivirus*, species: Gallid alphaherpesvirus2) (https://talk.ictvonline.org). MDV is the causative agent of Marek’s disease (MD), characterized by lymphomas and immunosuppression in infected chickens. Although MD caused severe economic losses to the poultry industry in the 1960s, the disease is currently controlled through vaccination using attenuated strains of the virus (1, 2). However, MDV field strains are constantly evolving toward higher virulence, leading to sporadic outbreaks of MD even in vaccinated flocks in some countries (3–8). Consequently, the potential for future outbreaks caused by highly virulent MDV strains is a matter of concern (9).

The life cycle of MDV in chickens consists of four main phases: early cytolytic phase, latent phase, late cytolytic phase, and proliferative phase (10). MDV infection starts with the inhalation of airborne cell-free virus particles into the respiratory tract (11). MDV also replicates in macrophages residing in the lungs of infected chickens (12). Macrophages are thought to deliver MDV to lymphoid tissues, such as the thymus, bursa of Fabricius, and spleen (1, 13). MDV infection is accompanied by semi-productive lytic replication in B cells, T cells, and NK cells that induces early and transient atrophy of the primary lymphoid organs (14–16). In addition to lytic replication, MDV establishes latent infection in CD4^+^ T cells, and only a few infected cells are subsequently transformed, resulting in the development of lymphomas in infected chickens (17). T cells account for most of the MDV-transformed tumor cells, indicating an oligoclonal expansion of transformed CD4^+^ T cells (17, 18).

*Meq*, an MDV oncogene, encodes a 339 amino-acid protein, Meq, that is expressed in both the lytic and latent phases (19). The *Meq*-deleted recombinant MDV (rMDV) failed to develop lymphomas in infected chickens, indicating that Meq plays an essential role in the transformation induced by virulent MDV (20). Meq is a basic leucine zipper (bZIP) transcription factor comprising an N-terminal domain that contains a basic region (required for binding to the target regions) and a ZIP motif (similar to those of the proto-oncoproteins Jun and Fos) and a transactivation domain that is characterized by proline-rich repeats (PRRs) in the C-terminus (19). Meq regulates the expression of target genes by forming a homodimer with itself or heterodimers with AP-1 family proteins, such as c-Jun and Fos, through ZIP (21). Meq upregulated the genes involved in the *v-Jun* transforming pathway, such as *JTAP-1*, *JAC*, and *HB-EGF*, in DF-1 cells (22). Additionally, Meq upregulated the expression of *bcl-2* and *ski* and downregulated the expression of *dap5* and *fas* in DF-1 cells, consistent with its anti-apoptotic properties (22, 23). Furthermore, Meq interacts with the tumor suppressor p53, inhibiting its functionality (24). Thus, Meq exhibits diverse functions, including transcriptional regulation, by binding to a variety of proteins related to tumorigenesis.

The diversity in Meq among MDV strains with varying virulence has been considered a potential factor influencing MDV virulence (25). Notably, the sequences of several amino acid residues in the basic region and PRRs are shared among highly virulent strains, and the introduction of amino acid substitutions in these regions affects Meq transactivation activity (26–28). Additionally, the difference in the amino acid sequences of Meq altered the cellular composition of tumor lesions in chickens infected with the recombinant MDV (29). Moreover, experimental infection using rMDVs revealed that rMDV harboring Meq from a highly virulent strain, RB-1B, exhibited higher virulence than those harboring Meq from low-virulence strains (30). Thus, the amino acid sequences of Meq are largely associated with the virulence and pathology of MDV.

In addition to polymorphisms, insertion in the transactivation domain of Meq has been detected in several MDV strains (25, 31). A long isoform of Meq, known as L-Meq, is characterized by a 59/60-amino-acid insertion in the transactivation domain that increases the number of PRRs. L-Meq was first discovered in CVI988/Rispens, an attenuated vaccine strain (31), and subsequently identified in low-virulent strains isolated in the USA (25). Therefore, L-Meq was initially assumed to contribute to the low virulence of MDV. However, further investigations have revealed that highly virulent MDV strains circulating in Australia express L-Meq with polymorphisms in PRRs (32). We previously analyzed the effect of the insertion in Meq on the activity of the meq promoter and revealed that it enhanced meq promoter-mediated transcription (33). Additionally, we clarified that the RB1B-based rMDV encoding L-Meq (vRB-1B_L-Meq) caused higher mortality and tumor incidence than rMDV encoding Meq (vRB-1B_Meq) in infected chickens (33). However, the detailed mechanisms by which the insertion in Meq alters MDV virulence are yet to be determined.

Here, we investigated the impact of the insertion in Meq on the process of MDV pathogenesis. To assess the effects of this insertion on the functions of Meq, we compared the transcriptional regulation ability of Meq and L-Meq using reporter assays. To examine the differences in the patterns of gene expression by each Meq isoform, we performed RNA-seq analysis on tumor lesions in chickens experimentally infected with vRB-1B_Meq and vRB-1B_L-Meq. Finally, we compared the dynamics of transformed T cells and T cell subsets involved in immune response between vRB-1B_Meq- and vRB-1B_L-Meq-infected chickens.

## RESULTS

### Amino acid insertion in Meq enhances its transactivation activity

**Fig 1:**
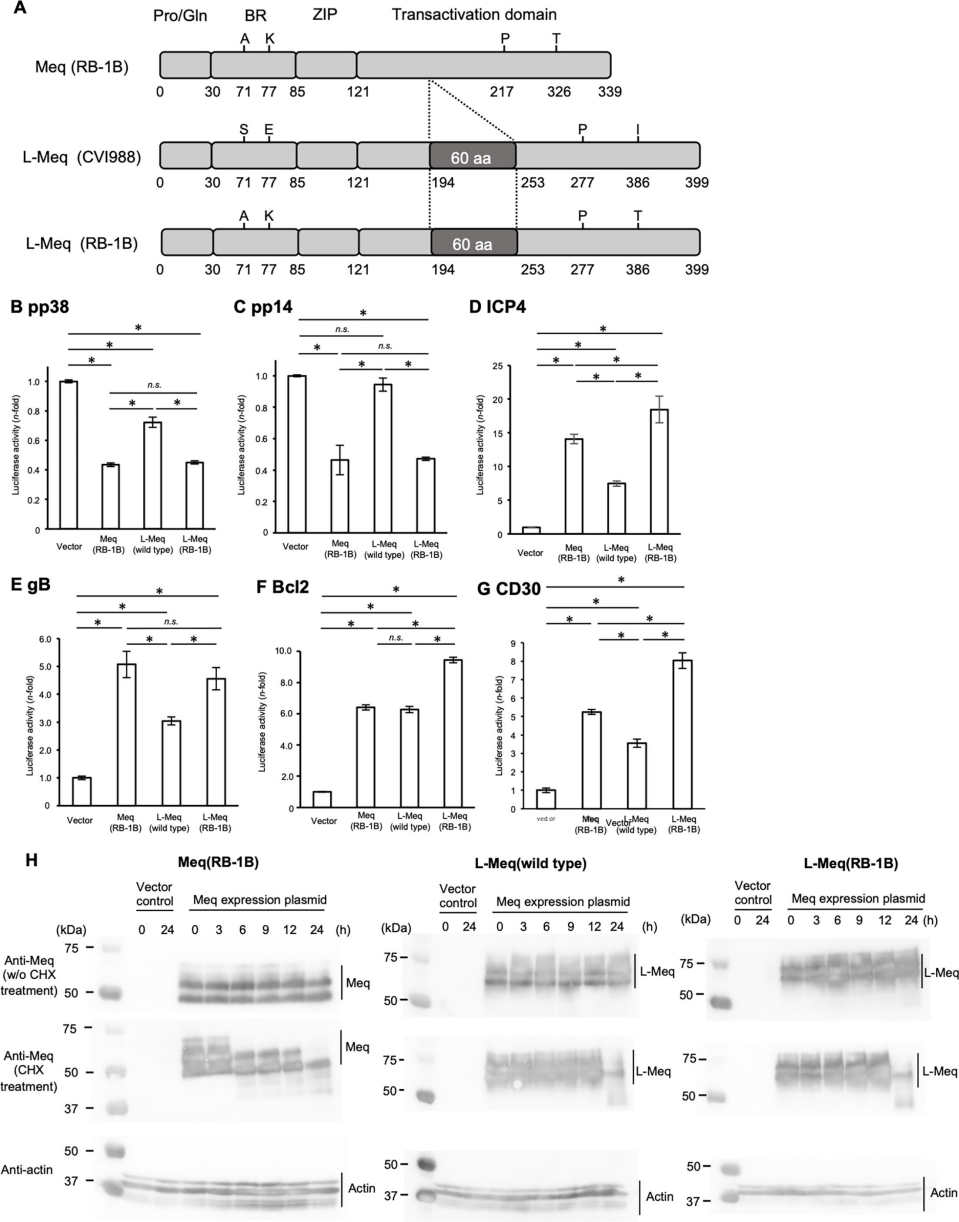
Analysis of transcriptional regulation by the Meq and L-Meq proteins. (**A**) Schematic representation of the Meq isoforms. The structures of Meq from RB-1B, long-Meq isoform (L-Meq) from CVI988, and L-Meq (RB-1B) whose sequences were matched with those of RB-1B-Meq, except for the insertion in the transactivation domain. Meq comprises a proline/glutamine (Pro/Gln)-rich domain followed by a basic region (BR) and leucine zipper (ZIP) at the N-terminal region and a transactivation domain at the C-terminal region. The Meq isoforms include amino acid polymorphisms in the BR and transactivation domain. L-Meq is characterized by a 60-amino-acid insertion in the transactivation domain. (**B and C**) Transrepression effects of the Meq isoforms. The transrepression effects of RB-1B-Meq, wild-type L-Meq (CVI988), and L-Meq (RB-1B) on (**B**) the pp38 promoter and (**C**) the pp14 promoter-driven luciferase activities were compared. DF-1 cells in each well were transfected with 300 ng of expression plasmids for each Meq isoform, 500 ng of reporter plasmid, and 5 ng of control pRL-TK plasmid. Luciferase activities were analyzed 24 h post-transfection. Firefly luciferase activity is expressed relative to the mean basal activity in the presence of pCI-neo after normalization to *Renilla* luciferase activity. (D–G) Transactivation activities of the Meq isoforms. The transactivation activities of RB-1B-Meq, wild-type L-Meq (CVI988), and L-Meq (RB-1B) on (**D**) the icp4 promoter, (**E**) the gb promoter, (**F**) the bcl-2 promoter, and (**G**) the cd30 promoter-driven luciferase activities were compared. DF-1 cells in each well were transfected with 300 ng of expression plasmids for each Meq isoform, 200 ng of c-Jun expression plasmid, 500 ng of reporter plasmid, and 5 ng of control pRL-TK plasmid. Error bars indicate standard deviations. Three independent experiments were performed in triplicate. **P < 0.01, Tukey’s multiple comparison test. (**H**) Stability of Meq isoforms. The stability of RB-1B-Meq, wild-type L-Meq (CVI988), and L-Meq (RB-1B) was compared. DF-1 cells in each well were transfected with 150 ng of expression plasmids for each Meq isoform. The cells in each well were added 100 µg/mL of cycloheximide 24 h after transfection. The transfected cells were electrophoretically separated in 10% SDS-PAGE and then transferred onto a nitrocellulose membrane. The membrane was incubated with the rabbit anti-Meq antibody (20  µg/mL) and mouse anti-chicken actin antibody (20  µg/mL) for 1  h, and then incubated with anti-rabbit IgG secondary antibody conjugated with HRP or anti-mouse IgG_1_ secondary antibody conjugated with HRP for 30  min, respectively. Three independent experiments were performed in triplicate.

We previously reported that insertion in the transactivation domain of Meq enhanced the transactivation activity of the meq promoter (33). Therefore, we further investigated the effects of this insertion on the transcriptional regulation by Meq on other promoter regions whose activities are regulated by Meq. To assess the effect of insertion on transcriptional regulation, we introduced mutations in L-*Meq* of CVI988 and manipulated its sequence to be identical to that of RB-1B-*Meq*, except for the insertion (Fig. 1A). The resultant expression plasmids were termed Meq (RB-1B), L-Meq (wild-type), and L-Meq (RB-1B).

Next, we analyzed the effects of Meq on the activities of pp38, pp14, infected-cell protein 4 (icp4), and glycoprotein (gb) promoters in the MDV genome and the bcl-2 and cd30 promoters in the chicken genome. Meq/Meq homodimers reportedly suppressed the activities of pp38 and pp14 promoters (34). Therefore, we examined the difference in the transrepression on pp38 and pp14 promoters among Meq and L-Meq constructs. Although all Meq isoforms exerted transrepression effects on the pp38 promoter, the transrepression by L-Meq (wild-type) was significantly weaker than that by Meq (RB-1B) or L-Meq (RB-1B), whereas Meq (RB-1B) and L-Meq (RB-1B) exhibited similar levels of suppression (Fig. 1B). In addition, a similar tendency of transrepression by L-Meq (wild-type) was observed on the pp14 promoter (Fig. 1C). These data suggest that although the differences in certain amino acid residues affect Meq-mediated transrepression, the insertion in Meq does not alter its transrepression of pp38 and pp14 promoter activities.

Given that Meq/c-Jun heterodimer upregulates the transcriptional activities of the promoters of some viral genes such as *icp4* and *gb* and host genes such as *bcl-2* and *cd30* (22, 29, 35), we compared the transactivation activities of Meq and L-Meq constructs on these promoters. L-Meq (wild-type) demonstrated lower transactivation activity than that of Meq (RB-1B) on most promoters, except the bcl-2 promoter. On the contrary, L-Meq (RB-1B) exhibited significantly higher activity on the icp4, bcl-2, and cd30 promoters than Meq (RB-1B), whereas the transactivation activity of L-Meq (RB-1B) was similar to that of Meq (RB-1B) on gb promoter (Fig. 1D–G). Taken together, the insertion in Meq did not affect transrepression but increased transactivation activity on some promoters, although the effects of the insertion on its transactivation activity appear to vary for different promoters.

### Effect of insertion in Meq on protein stability

As the stability of some viral proteins is increased by the insertion of proline-rich sequences (36), we examined the effects of insertion in Meq on its protein stability. Some signals were observed at approximately 50 kDa in DF-1 cells expressing Meq (RB-1B) until 3 h after the treatment with cycloheximide, presumably due to post-translational modifications; however, signals higher than 50 kDa disappeared at 6 h post-treatment (Fig. 1H and Fig. S1). On the contrary, the signals showing the highest molecular weight observed at 0 h in cells expressing L-Meq (wild-type) and L-Meq (RB-1B) were detectable up to 12 h post-treatment (Fig. 1 and Fig. S1). These findings suggest that the insertion enhances the stability of Meq, thereby enhancing its transactivation activity.

### Recombinant viruses and their growth kinetics *in vitro*

**Fig 2:**
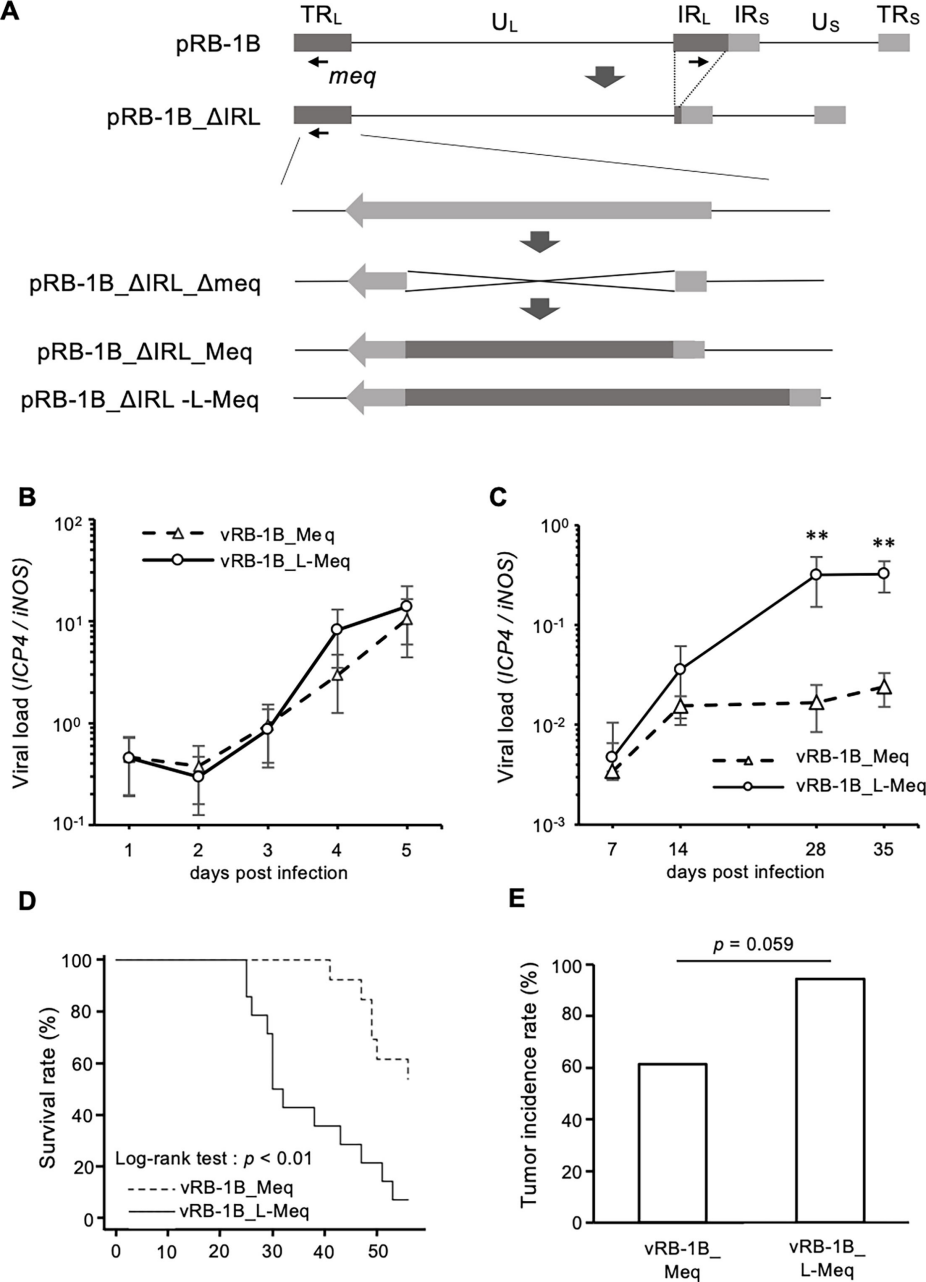
Reconstitution of recombinant Marek’s disease viruses (rMDVs) and their characterization *in vivo*. (**A**) Schematic diagrams of the constructs cloned using the RB-1B genome in this study. In the RB-1B genome cloned as the bacterial artificial chromosome (BAC) plasmid (pRB-1B), most of the internal repeat long (IRL) regions were deleted in this plasmid, designated as pRB-1B_ΔIRL, and used for mutagenesis. *Meq* in the terminal repeat long (TRL) was replaced with RB-1B-*Meq* or L-*Meq* (RB-1B) (encoding the long-Meq containing the insertion) by two-step red-mediated mutagenesis. (**B**) CEFs were infected with 50 pfu of rMDVs. The infected cells were collected daily for 6 days. The viral loads in the infected cells were analyzed by quantitative PCR (qPCR) (7–35 dpi: control group: *n* = 4, vRB-1B_Meq-infected group: *n* = 4, vRB-1B_L-Meq-infected group: *n* = 4). Three independent experiments were performed in triplicate. Error bars indicate standard deviations. (**C**) The viral loads in the whole blood of chickens infected with vRB-1B_Meq and vRB-1B_L-Meq were determined by quantitative polymerase chain reaction (qPCR). Error bars indicate standard deviations. **P < 0.01, Mann–Whitney U test. (**D**) Survival rate in chickens infected with rMDVs in the experimental infection. The survival rate among the groups was analyzed using the Log-rank test. (**E**) Tumor incidence in chickens infected with rMDVs during the study period. *P < 0.05, Fisher’s exact test.

We investigated the mechanisms of enhanced virulence induced by the insertion in Meq by generating RB-1B-based rMDVs encoding RB-1B-Meq or L-Meq (RB-1B). We inserted RB-1B-*Meq* and L-*Meq* (RB-1B) into the internal repeat long (IRL)-deleted RB-1B genome cloned as a bacterial artificial chromosome (BAC) plasmid to replace native *Meq* in the terminal repeat long (TRL) (Fig. 2A). The resulting BAC plasmids were transfected into chicken embryo fibroblasts (CEFs), and IRL restoration in the reconstituted viruses, referred to as vRB-1B_Meq and vRB-1B_L-Meq, was confirmed by PCR (data not shown) and whole genome sequencing (BioSample accessions: LC849254, LC849255). No significant differences were observed in the *in vitro* growth kinetics among the rMDVs (Fig. 2B).

### *In vivo* replication of rMDVs and tumor incidence

We investigated the growth kinetics of rMDVs *in vivo* and the associated tumor incidence by infecting 1-day-old chicks with each rMDV. Although all the rMDVs exhibited efficient replication in the infected birds, vRB-1B_L-Meq showed significantly higher viral loads at 28 and 35 dpi compared with vRB-1B_Meq (Fig. 2C). Consistent with the results of our previous study (33), vRB-1B_L-Meq showed higher mortality than vRB-1B_Meq (*P* < 0.01; Fig. 2D) and tended to exhibit higher tumor incidence than vRB-1B_Meq (*P* = 0.059; Fig. 2E).

### Dynamics of major T-cell subsets in chickens infected with rMDVs

**Fig 3:**
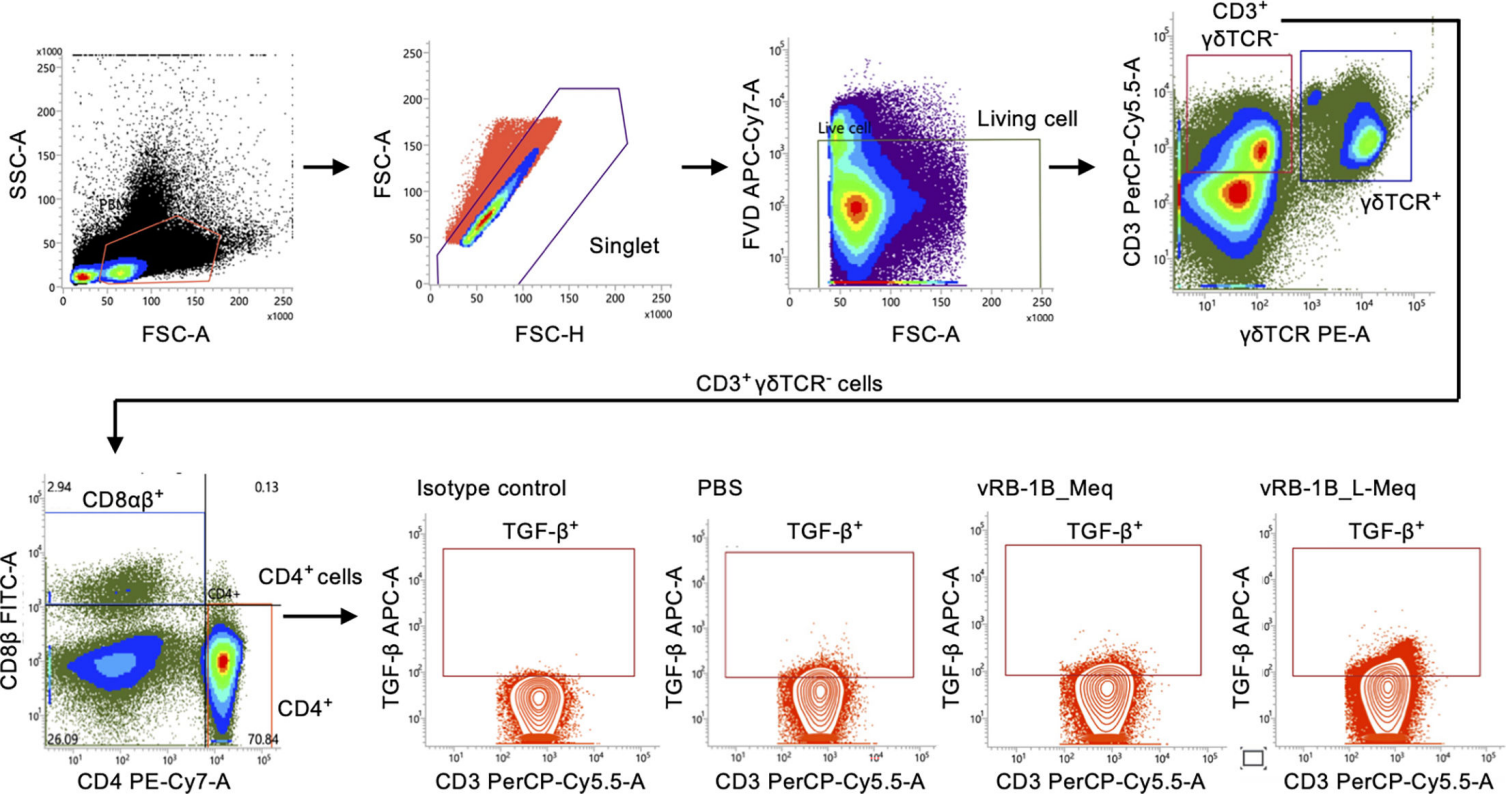
Gating strategy for the analysis of the dynamics of major T-cell subsets in chickens infected with rMDVs. A representative gating strategy is shown for analyzing the dynamics of γδ T cells, CD8αβ^+^ T cells, CD4^+^ T cells, and mTGF-β^+^ CD4^+^ T cells. Dead cells were excluded using Fixable Viability Dye eFluor780.

**Fig 4:**
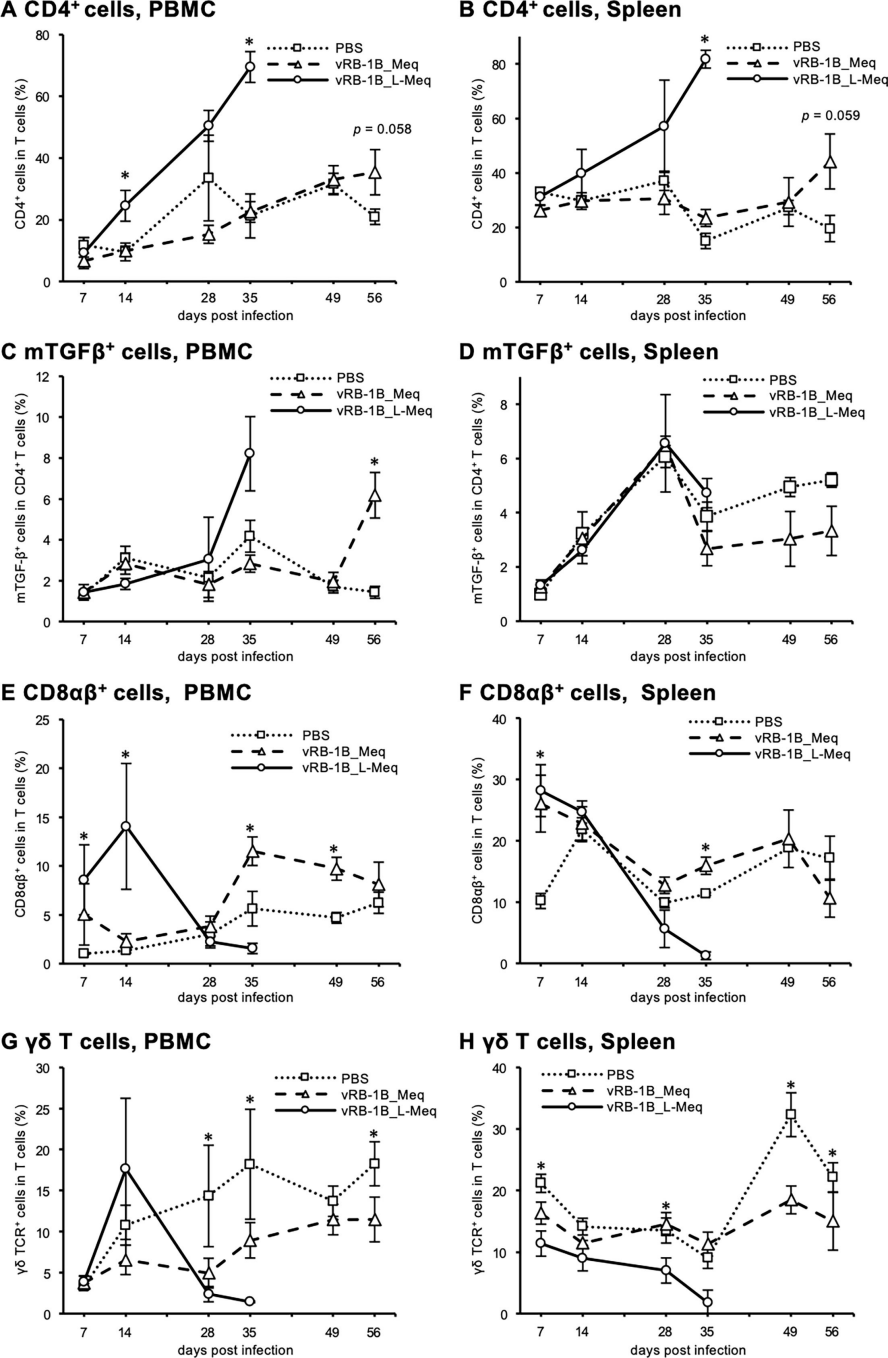
Dynamics of major T cell subsets in chickens infected with rMDVs. The dynamics of CD4^+^ cells, mTGF-β^+^ CD4^+^ cells, CD8αβ^+^ cells, and γδ T cells in T cells from PBMCs and spleens of chickens infected with vRB-1B-Meq or vRB-1B-L-Meq at each time point during the experimental period (7–49 dpi: control group: *n* = 4, vRB-1B_Meq-infected group: *n* = 4, vRB-1B_L-Meq-infected group: *n* = 4, 56 dpi: control group: *n* = 4, vRB-1B_Meq-infected group: *n* = 7). The percentages of CD4^+^ cells in the T cell population from (**A**) PBMCs and (**B**) spleens, mTGF-β^＋^ cells in the CD4^+^ T cell population from (**C**) PBMCs and (**D**) spleens, CD8αβ^+^ cells in the T cell population from (**E**) PBMCs and (**F**) spleens, and γδTCR^+^ cells in the T cell population from (**G**) PBMCs and (**H**) spleens were analyzed. Error bars indicate standard deviations. *P < 0.05, Kruskal–Wallis test (7–35 dpi), Mann–Whitney U test (49–56 dpi).

To compare the disease progression between the infected groups, we investigated the relative increase in CD4^+^ T cells and the associated decrease in other T-cell subsets in peripheral blood mononuclear cells (PBMCs) and spleens. The gating strategy for the analyses is illustrated in Fig. 3. The increase in the percentage of CD4^+^ T cells within T cells in both PBMCs and spleens of chickens infected with vRB-1B_L-Meq was earlier than those in the other groups, and the percentage was significantly higher at 35 dpi (Fig. 4A and B). Furthermore, the percentages of CD4^+^ T cells among T cells in both PBMCs and spleens of chickens infected with vRB-1B_Meq tended to be higher than that of the control group at 56 dpi (*P* = 0.058 and *P* = 0.059, respectively; Fig. 4A and B).

Chickens with MD had an elevated percentage of Treg-like cells, which are characterized by the expression of membrane-bound transforming growth factor-β (mTGF-β) and CD4 (37). In this study, we observed an increase in the percentage of mTGF-β^+^ cells in the CD4^+^ T cell population in PBMCs of chickens infected with rMDVs. Notably, the percentage of mTGF-β^+^ cells in the CD4^+^ T cell population in PBMCs of the vRB-1B_L-Meq-challenged group tended to increase at 35 dpi, whereas the vRB-1B_Meq-challenged group showed a delayed increase at 56 dpi (Fig. 4C). However, no significant difference among the groups was observed in the percentage of mTGF-β^+^ cells in the spleens of chickens (Fig. 4D).

We also analyzed the dynamics of CD8αβ^+^ T cells, critical mediators of cellular immunity against MDV-infected cells. In chickens infected with vRB-1B_L-Meq, the percentage of CD8αβ^+^ T cells within the T cell population in PBMCs was significantly increased at 7 and 14 dpi but decreased at 35 dpi (Fig. 4E), in contrast to the increase in the percentage of CD4^+^ T cells in the T cell populations in PBMCs of vRB-1B_L-Meq-infected chickens (Fig. 4A and D). On the contrary, the percentage of CD8αβ^+^ T cells within the T-cell population in PBMCs of vRB-1B_Meq-infected chickens increased at 35 and 49 dpi but did not differ from that of the control group at 56 dpi (Fig. 4E). In the spleens, the percentage of CD8αβ^+^ T cells in the T cell population in both infected groups increased at 7 dpi but decreased at 35 dpi in the vRB-1B_L-Meq-infected group and tended to decrease at 56 dpi in the vRB-1B_Meq-infected group (Fig. 4F).

Next, we examined the percentage of γδ T cells in chickens infected with rMDVs, given that γδ T cells have protective functions against MD in chickens vaccinated with CVI988 (38, 39). The percentage of γδ T cells among the T cell population in PBMCs of chickens decreased at 28 and 35 dpi in the vRB-1B_L-Meq-infected group, and it was consistently lower in the vRB-1B_Meq-infected group than in the control group (Fig. 4G). Additionally, the percentages of γδ T cells within the T cell population in the spleens of chickens infected with vRB-1B_L-Meq and vRB-1B_Meq tended to be lower than that of the control group throughout the experimental period (Fig. 4H). Thus, the CD8αβ^+^ T cells and γδ T cells failed to suppress the proliferation of transformed CD4^+^ T cells. Taken together, T cell subsets in the terminal phase of infection in each group showed similar dynamics, but the vRB-1B_L-Meq-infected group showed it earlier.

### Dynamics of CD8α^+^ γδ T-cell subsets in chickens infected with rMDVs

**Fig 5:**
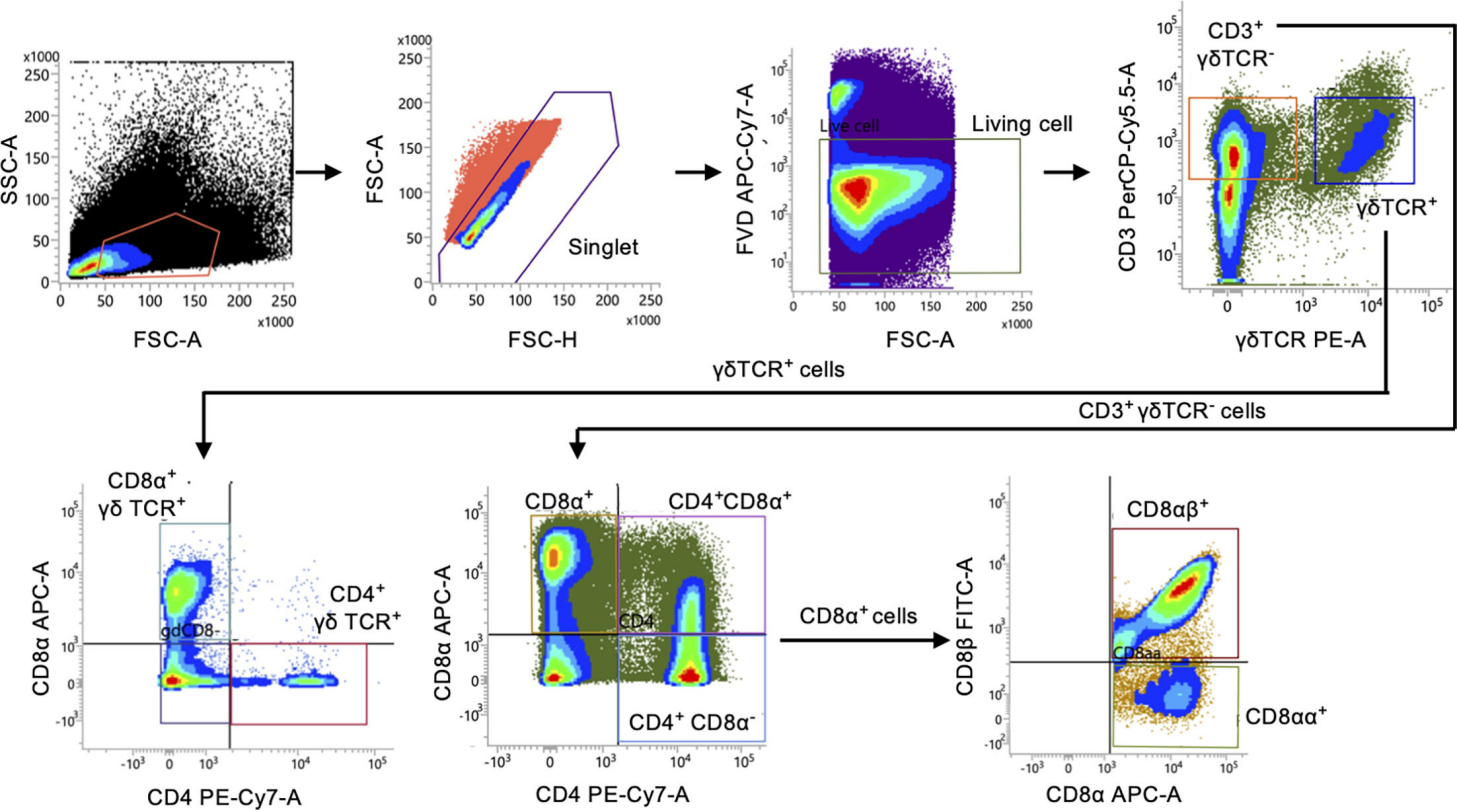
Gating strategy for the analysis of the dynamics of T-cell subsets involved in immune response in chickens infected with rMDVs. A representative gating strategy is shown for analyzing the dynamics of CD8α^+^ γδ T cells, CD4^+^ γδ T cells, and CD8αα^+^ T cells. Dead cells were excluded using Fixable Viability Dye eFluor780.

**Fig 6:**
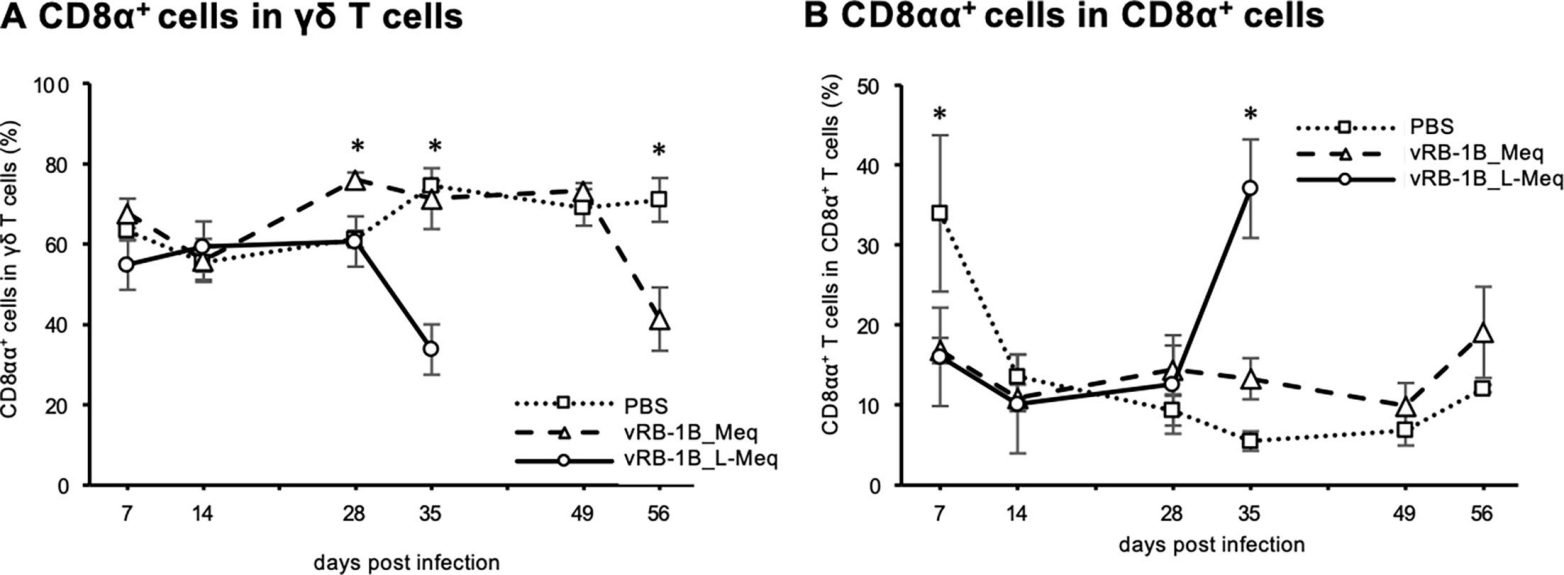
Dynamics of T-cell subsets involved in immune response in chickens infected with rMDVs. The dynamics of CD8α^+^ γδ T cells and CD8αα^+^ T cells from spleens of chickens infected with vRB-1B-Meq or vRB-1B-L-Meq at each time point during the experimental period (7–49 dpi: control group: *n* = 4, vRB-1B_Meq-infected group: *n* = 4, vRB-1B_L-Meq-infected group: *n* = 4, 56 dpi: control group: *n* = 4, vRB-1B_Meq-infected group: *n* = 7). (**A**) The percentages of CD8α^+^ T cells in the γδ T cell population from spleens, (**B**) the percentage of CD8αα^+^ cells in CD8α^+^ T cell population from spleens, and (**C**) the percentage of CD4^+^CD8α^-^ T cells in CD4^+^ T cell population from spleens were analyzed. Error bars indicate standard deviations. *P < 0.05, Kruskal–Wallis test (7–35 dpi), Mann–Whitney U test (49–56 dpi).

CD8α^+^ γδ T cells (TCRγδ^+^CD3^+^CD4^-^CD8α^+^) were increased by vaccination with CVI988 and expressed IFN-γ, suggesting the contribution to Th1 polarization and cellular immunity (38, 40). Therefore, we analyzed the dynamics of CD8α^+^ γδ T cells between the infected groups, based on the gating strategy illustrated in Fig. 5. Our data showed that the percentage of CD8α^+^ γδ T cells within the γδ T cells in spleens of vRB-1B_Meq-infected chickens was significantly increased at 28 dpi, compared with those in the control and vRB-1B_L-Meq-infected groups, but decreased at 56 dpi (Fig. 6A). In contrast, the percentage of CD8α^+^ γδ T cells among the γδ T cell population in the spleen of rRB-1B_L-Meq-infected chickens did not differ from that of the control group until 28 dpi but was significantly reduced at 35 dpi (Fig. 6A).

### Dynamics of CD8αα^+^ T cells in chickens infected with rMDVs

A previous study showed that CD8αα^+^ T cells (TCRγδ^−^CD3^+^CD8α^+^CD8β^-^) are more abundant in MD-resistant chickens and increase during the proliferation phase (40). In the present study, the percentages of CD8αα^+^ T cells among CD8α^+^ T cells in spleens in both infected groups were significantly lower than that in the control group at 7 dpi (Fig. 6B). In contrast, a significant increase in CD8αα^+^ T cells in the CD8α^+^ T cell population was observed in the spleens of the vRB-1B_L-Meq-infected chickens at 35 dpi (Fig. 6B), suggesting that CD8αα^+^ T cells responded to viral antigens or transformed cells.

### Composition of T cell subsets and transcriptome analysis of tumor lesions

**Fig 7:**
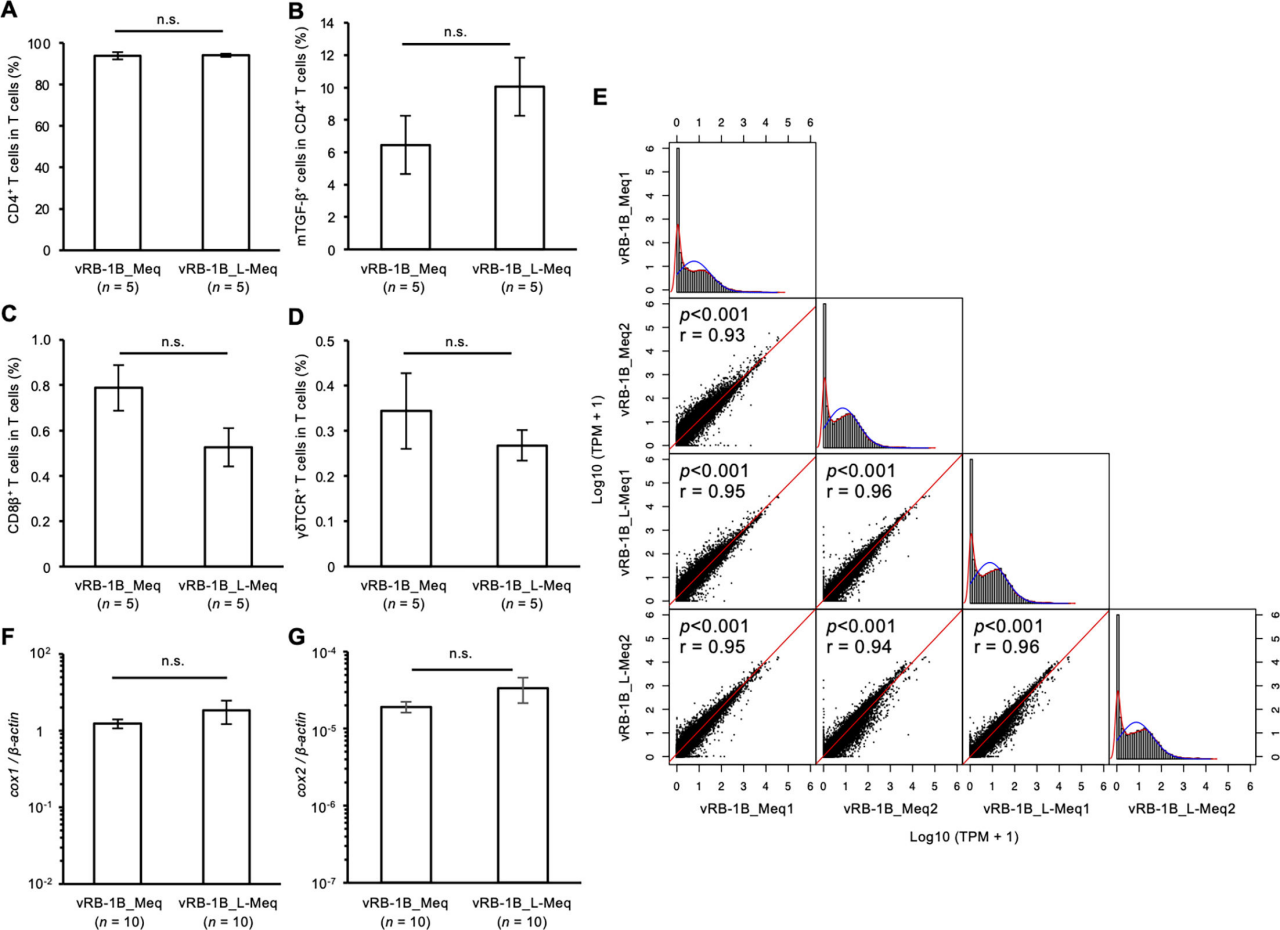
Composition of T cell subsets and transcriptome analysis of tumor lesions of rMDV-infected chickens. (A–D) The composition of T cell subsets in tumor lesions from chickens that developed tumors in the kidneys or gonads during the experimental period was analyzed by flow cytometry (vRB-1B_Meq-infected group: *n* = 5, vRB-1B_L-Meq-infected group: *n* = 5). (**A**) The percentages of CD4^+^ cells in the T cell population, (**B**) mTGF-β^+^ cells in the CD4^+^ T cell population, (**C**) CD8αβ^+^ cells in the T cell population, and (**D**) γδTCR^+^ cells in the T cell population were analyzed. (**E**) The scatter plots show the Spearman rank correlation between each tumor lesion from chickens infected with vRB-1B-Meq and vRB-1B-L-Meq. r: Spearman’s rank correlation coefficient. The expression levels of (**F**) *cox1* and (**G**) *cox2* in tumor lesions from chickens infected with vRB-1B-Meq or vRB-1B-L-Meq at 7 dpi were determined by qPCR.

A previous report showed that the cellular composition in tumor lesions was altered by the amino acid sequence of Meq in the recombinant MDV (41). Therefore, we compared the cellular composition in five solid tumors between the vRB-1B_Meq- and vRB-1B_L-Meq-infected groups; however, no difference in the proportions of total T cells (CD3^+^ cells), B cells (Bu-1^+^ cells), and macrophages was observed (data not shown). Additionally, the T cell population in all tumor lesions in both infected groups was composed of approximately 95% of CD4^+^ T cells (Fig. 7A), including a phenotype expressing mTGF-β (Fig. 7B), and the percentages of CD8αβ^＋^ T cells and γδ T cells were less than 1% (Fig. 7C and D). No significant difference in these T cell subsets between the infected groups was observed (Fig. 7A through D). Thus, the insertion in Meq did not affect the cellular composition in tumor lesions.

Next, we compared the transcriptome in tumor lesions of vRB-1B_Meq- and vRB-1B_L-Meq-infected chickens using RNA-seq. Of the 39,088 transcripts obtained, 15,967 transcripts (40.8%) indicated expression intensities, of which log2 transcript per million (TPM) was more than 0 in both infected groups. Of them, 15,262 and 14,601 transcripts were highly expressed in vRB-1B_L-Meq and vRB-1B_Meq samples, respectively. The scatter plots showing the Spearman rank correlation display significant positive correlations in all comparisons between samples (Fig. 7E), suggesting that the patterns of gene expression in tumor lesions of chickens infected with vRB-1B_L-Meq and vRB-1B_Meq were largely identical. We further analyzed the expression levels of cyclooxygenase-1 (*cox1*) and *cox2* genes, which showed increased expression in tumor lesions from both groups in the RNA-seq data; this observation confirmed no significant differences in *cox1* and *cox2* expression levels in the 10 other tumor lesion samples each from vRB-1B_Meq and vRB-1B_L-Meq groups (Fig. 7F and G). Collectively, the transformation by both rMDVs appeared to be regulated by similar pathways or by modulating the expression of similar target genes.

In our RNA-seq data, 10,313 transcripts (26.4%) were annotated with Entrez gene IDs and showed detectable expression levels (log₂ TPM > 0). Among them, 2,132 transcripts exhibited more than 2-fold higher expression intensities in vRB-1B_L-Meq samples than those in vRB-1B_Meq samples; in contrast, 906 transcripts showed a more than 2-fold higher expression in vRB-1B_Meq samples, biological processes (BP), cellular components (CC), and molecular functions (MF). Subsequently, GO analysis was conducted on these differentially expressed genes, classified as categorized under biological processes (BP), cellular components (CC), and molecular functions (MF). The most significantly enriched GO terms in each category are shown in Tables 1 to 3, in order of their significance in terms of *P* value. Notably, in the BP category of vRB-1B_L-Meq samples, genes with GO terms associated with “T cell proliferation,” such as *il15*, *il21*, and *cd28*, were enriched, suggesting an enhanced proliferative potential in cells transformed by vRB-1B_L-Meq. Furthermore, genes related to “chemotaxis,” such as *cxcr5*, *ccl4*, and *ccl13*, were identified in the BP and MF categories of vRB-1B_L-Meq. In contrast, in the vRB-1B_Meq samples, GO terms related to metabolism—particularly lipid metabolism—such as “cellular lipid metabolic process,” “cholesterol homeostasis,” and “triglyceride homeostasis” were enriched in the BP category. Moreover, synthetic pathways of bioactive substances derived from lipids, such as the “prostaglandin biosynthetic process” and “estrogen biosynthetic process,” were also upregulated.

**TABLE 1:** Top 20 Gene Ontology categories with respect to “Biological Process”

	L-Meq			Meq		
	*P*-value	GO term	Functional category	*P*-value	GO term	Functional category
#1	2.10E-04	GO:0042102	Positive regulation of T cell proliferation	3.50E-04	GO:0001516	Prostaglandin biosynthetic process
#2	7.30E-04	GO:0006955	Immune response	8.10E-03	GO:0045741	Positive regulation of epidermal growth factor-activated receptor activity
#3	1.20E-03	GO:0006954	Inflammatory response	1.00E-02	GO:1901224	Positive regulation of NIK/NF-kappaB signaling
#4	1.40E-03	GO:0071347	Cellular response to interleukin 1	1.30E-02	GO:0007173	Epidermal growth factor receptor signaling pathway
#5	2.40E-03	GO:0070098	Chemokine-mediated signaling pathway	1.30E-02	GO:1903078	Positive regulation of protein localization to plasma membrane
#6	2.70E-03	GO:0006915	Apoptotic process	1.30E-02	GO:0000226	Microtubule cytoskeleton organization
#7	4.20E-03	GO:0071356	Cellular response to tumor necrosis factor	1.40E-02	GO:0034145	Positive regulation of toll-like receptor 4 signaling pathway
#8	5.70E-03	GO:0030198	Extracellular matrix organization	1.40E-02	GO:0006703	Estrogen biosynthetic process
#9	7.30E-03	GO:0002755	MyD88-dependent toll-like receptor signaling pathway	1.50E-02	GO:0032722	Positive regulation of chemokine production
#10	8.50E-03	GO:0007596	Blood coagulation	1.70E-02	GO:0042632	Cholesterol homeostasis
#11	1.30E-02	GO:0035556	Intracellular signal transduction	2.20E-02	GO:0046628	Positive regulation of insulin receptor signaling pathway
#12	1.50E-02	GO:0048247	Lymphocyte chemotaxis	2.50E-02	GO:1990830	Cellular response to leukemia inhibitory factor
#13	1.50E-02	GO:0009968	Negative regulation of signal transduction	3.50E-02	GO:0032731	Positive regulation of interleukin-1 beta production
#14	1.60E-02	GO:0007155	Cell adhesion	4.40E-02	GO:0070328	Triglyceride homeostasis
#15	2.00E-02	GO:0002062	Chondrocyte differentiation	4.40E-02	GO:0045840	Positive regulation of mitotic nuclear division
#16	2.10E-02	GO:0051491	Positive regulation of filopodium assembly	4.40E-02	GO:0061025	Membrane fusion
#17	2.10E-02	GO:0002040	Sprouting angiogenesis	5.60E-02	GO:0044255	Cellular lipid metabolic process
#18	2.10E-02	GO:0001525	Angiogenesis	6.50E-02	GO:0050729	Positive regulation of inflammatory response
#19	2.50E-02	GO:0002224	Toll-like receptor signaling pathway	6.70E-02	GO:0008284	Positive regulation of cell proliferation
#20	2.80E-02	GO:0002548	Monocyte chemotaxis	7.00E-02	GO:0007098	Centrosome cycle

**TABLE 2:** Top 5 Gene Ontology categories with respect to “cellular component”

	L-Meq			Meq		
	*P*-value	GO term	Functional category	*P*-value	GO term	Functional category
#1	9.70E-05	GO:0005615	Extracellular space	1.50E-03	GO:0005576	Extracellular region
#2	2.50E-03	GO:0031012	Extracellular matrix	1.90E-03	GO:0005737	Cytoplasm
#3	2.70E-03	GO:0005581	Collagen trimer	8.20E-03	GO:0045095	Keratin filament
#4	4.00E-03	GO:0009897	External side of plasma membrane	8.70E-03	GO:0005615	Extracellular space
#5	7.00E-03	GO:0005737	Cytoplasm	1.60E-02	GO:0043235	Receptor complex

**TABLE 3:** Top 5 Gene Ontology categories with respect to “molecular function”

L-Meq			Meq		
*P*-value	GO term	Functional category	*P*-value	GO term	Functional category
3.30E-04	GO:0008009	Chemokine activity	1.40E-03	GO:0005154	Epidermal growth factor receptor binding
2.00E-03	GO:0005524	ATP binding	4.90E-03	GO:0004601	Peroxidase activity
3.10E-03	GO:0003953	NAD+ nucleosidase activity	1.30E-02	GO:0005080	Protein kinase C binding
5.10E-03	GO:0050135	NAD(P)+ nucleosidase activity	1.50E-02	GO:0004862	cAMP-dependent protein kinase inhibitor activity
5.10E-03	GO:0061809	NAD+ nucleotidase, cyclic ADP-ribose generating	1.50E-02	GO:0003953	NAD+ nucleosidase activity

## DISCUSSION

In the present study, we examined the impact of the insertion in Meq on virulence by analyzing Meq-mediated transcriptional regulation and T-cell dynamics in rMDV-infected chickens. We found that the insertion enhanced the stability of Meq and its transactivation activity on the bcl-2, icp4, and cd30 promoters. Therefore, the insertion in Meq may more robustly promote the expression of transformation-related genes in latently infected cells. Supporting this hypothesis, we observed that CD4^+^ T cells, including an MDV-transformed cell phenotype (mTGF-β^+^CD8α^−^), increased earlier in vRB-1B_L-Meq-infected chickens. These findings suggest that insertion in Meq facilitates the early transformation of CD4^+^ T cells by enhancing the expression levels of tumor-associated genes, thereby accelerating MDV-mediated tumorigenesis.

The insertion in Meq enhanced the transactivation activity on bcl-2, cd30, and icp4 promoters as well as meq promoter (33); this is presumably due in part to the enhanced protein stability by the insertion in Meq. Epstein–Barr virus (EBV), an oncogenic herpesvirus, encodes EBV nuclear antigen 1 (EBNA1), which is consistently expressed in all EBV-associated malignancies, and the proline-alanine repeat sequence in EBNA1 inhibits ATP-dependent ubiquitination and proteasomal degradation (36). Moreover, the insertion of this proline-rich sequence in another viral protein, EBNA4, prevented its degradation via the proteasome. Therefore, an increase in the PRRs in L-Meq may be involved in the inhibition of proteasomal degradation, resulting in the enhanced protein stability of Meq. Additionally, the insertion in Meq may affect the protein structure, thereby enhancing its stability. Notably, Meq-derived peptides can induce IFN-γ-producing MDV-specific CD8^+^ T cells (42); therefore, the enhanced protein stability due to the insertion may impair the efficiency of antigen processing and presentation, facilitating immune evasion of the transformed cells. Further analysis, including the effects of the number of PRRs on proteasomal degradation and protein structure, is warranted to clarify the relationship between the stability of Meq and MDV virulence.

In contrast, the insertion in Meq did not enhance the activity of the gb promoter, which is different from other promoters analyzed in this study. Meq activates gene expression by forming heterodimers with the AP-1 family protein and binding to the Meq response element 1 (MERE1) located within the promoter region (34). Although MERE1 sequences are present in the gb promoter region, the number of MERE1 sequences is lower than in the meq and icp4 promoters (34), potentially accounting for different effects by insertion on each promoter. Therefore, a comprehensive analysis, including the interaction between Meq and other transcriptional factors and the sequence information of their binding regions, is required to clarify the effects of the insertion on transcriptional regulation of Meq.

In this study, we observed an increase in the percentage of a Treg-like phenotype expressing mTGF-β and CD4 in PBMCs of MDV-infected chickens as previously reported (37), likely reflecting the expansion of lymphoma cells. Additionally, the proportion of CD4^+^ T cells within the T cell population in vRB-1B_L-Meq-infected chickens increased from 14 dpi. As MDV-transformed cells could emerge as early as 10 dpi (10), the increased CD4^+^ T cells in this early phase of infection may include transformed cells. Thus, our flow cytometry analysis suggests an earlier expansion of lymphoma cells in vRB-1B_L-Meq-infected chickens.

The proportion of CD8α^+^ γδ T cells increased after vaccination with CVI988 (40). Furthermore, the proportion of CD8α^+^ γδ T cells expressing IFN-γ was elevated during the early lytic phase in chickens superinfected with RB-1B after vaccination (38). These observations suggest that CD8α^+^ γδ T cells are involved in the immunity against MDV infection. However, in this study, the percentage of CD8α^+^ γδ T cells was decreased in both infected groups, coincident with an expansion of CD4^+^ T cells, suggesting a reduced response to transformed cells. Given that MDV-transformed Treg-like cells can suppress the proliferation of T cells through the production of prostaglandin E2 (37, 43), it is plausible that MDV-transformed cells also suppress the proliferation of CD8α^+^ γδ T cells. Notably, the decline in CD8α^+^ γδ T cells occurred earlier in vRB-1B_L-Meq-infected chickens, suggesting early disease progression in them.

An increase in the CD8αβ^+^ T cell population in PBMCs was observed at 14 dpi in the vRB-1B_L-Meq-infected group and later at 35 and 49 dpi in the vRB-1B_Meq-infected group. This elevation in CD8αβ^+^ T cells may reflect a response to MDV-transformed cells. The rise in CD4^+^ T cells followed the increase in CD8αβ^+^ T cells, as seen at 35 dpi in vRB-1B_L-Meq- and at 56 dpi in vRB-1B_Meq-infected groups. Furthermore, the percentage of CD8αα^+^ T cells was significantly increased in the vRB-1B_L-Meq-infected group at 35 dpi, likely in response to transformed cells. Previous studies showed that CD8αα^+^ T cells increased during the proliferation phase of MDV infection (44) and expanded rapidly after the secondary CVI988 vaccination (40), suggesting that CD8αα^+^ T cell population includes memory T cells against MDV. Thus, an increase in the percentage of CD8αα^+^ T cells in the late lytic phase observed in this study may have been caused by the reactivation of memory CD8αα^+^ T cells generated during the acute phase. Further functional analysis of CD8αα^+^ T cells may contribute to a more detailed understanding of MD pathogenesis. Importantly, neither CD8αβ^+^ T cells nor CD8αα^+^ T cells effectively eliminated transformed cells in the terminal phase of the disease, despite their proliferation.

The cellular composition significantly differed in tumor lesions caused by the RB-1B and Md5 strains (41). Furthermore, tumor lesions caused by the recombinant Md5 expressing RB-1B-Meq exhibited an intermediate cellular proportion between the two strains, suggesting that differences in the amino acid sequence of Meq can alter the cellular composition in tumor lesions. On the contrary, no significant differences were observed in total T cells, B cells, and macrophages, as well as each T cell subset in tumor lesions caused by vRB-1B_Meq and vRB-1B_L-Meq in this study. Thus, the 60-amino acid insertion in Meq appears to significantly accelerate disease progression without affecting the cellular composition in tumor tissues. Additionally, amino acid polymorphisms in Meq have the potential to possess mechanisms of enhanced virulence distinct from that caused by the insertion.

The RNA-seq data revealed that similar pathways were modulated by vRB-1B_Meq and vRB-1B_L-Meq in the process of transformation. However, GO analysis focusing on differences in the expression patterns between the groups revealed several transcriptional characteristics in tumor lesions of rMDV-infected chickens. In tumor lesions of vRB-1B_L-Meq-infected chickens, we observed predominant expression of genes related to immune response, including cytokines associated with lymphocyte proliferation, chemokines associated with lymphocyte migration, and genes associated with cell adhesion. The upregulation of these molecules may facilitate the proliferation of CD4^+^ T cells in the transformation phase and the migration and adhesion of tumor cells and immune cells, leading to the formation of solid tumors in various organs. In contrast, tumor lesions in chickens infected with vRB-1B_Meq showed predominant expression of genes involved in lipid metabolic pathways and cell proliferation. In particular, fatty acids such as palmitic acid are critical for MDV replication, and the fatty acid synthesis activated upon MDV infection promotes viral replication (45). Therefore, the tumor microenvironment in vRB-1B_Meq-infected chickens, where lipid metabolism is more active, may facilitate more frequent viral reactivation. Thus, there appear to be several differences in the characteristics of transformed cells between the groups. Additionally, a rapid disease progression in vRB-1B_L-Meq-infected chickens may affect the retention of features associated with the responses of immune cells, whereas tumor lesions in vRB-1B_Meq-infected chickens may reflect the properties of tumor cells.

For a more comprehensive understanding, further analysis is required to assess the contribution of the slight difference in gene expression to the virulence of MDV and to elucidate the events in the tumor microenvironment.

In the present study, we monitored the dynamics of CD4^+^ T cells, including both uninfected cells and tumor cells. Due to the lack of specific markers for MDV-transformed tumor cells, we could not differentiate tumor cells from the untransformed T-cell subpopulations in this study. We examined a method for flow cytometry analysis by intracellular staining using monoclonal Meq antibodies established by another group (46); this approach allowed more appropriate monitoring of tumor cells in the terminal phase of the disease. Additionally, other immune cell populations such as macrophages and NK cells would be required to further understand the differences in the pathogenesis caused by vRB-1B_Meq and vRB-1B_L-Meq. In this experiment, we used commercial chickens instead of SPF chickens. As commercial chickens are typically vaccinated against MD, early cytolytic infection and transformation of T-lymphocytes are significantly delayed in maternal antibody-positive chickens compared with those observed in SPF chickens (47). Therefore, the pathogenesis of MD observed in this study may progress more modestly than in SPF chickens. Moreover, commercially available chickens, including SPF chickens, are heterogeneous, which may result in high variability among individuals. Therefore, genetically homogeneous MD-susceptible chickens such as lines P and seven are preferable to evaluate MDV virulence. In this study, we confirmed that the virulence of each rMDV was comparable with that of our previous study (33) and observed a certain trend to accelerate the disease progression. However, conducting studies using genetically homogeneous MD-susceptible chickens would be required to further confirm the changes in the pathogenesis caused by the insertion in Meq.

The findings in this study have field relevance because CVI988 encodes L-Meq and shares the same insertion in its transactivation domain (48). The enhanced protein stability was observed in wild-type CVI988-L-Meq, similar to L-Meq (RB-1B); however, its transcriptional regulation ability was lower than those by L-Meq (RB-1B) and Meq (RB-1B). Additionally, a recombinant MDV expressing a Meq with polymorphisms in the basic region found in CVI988 exhibited significantly lower virulence than its parental strain (49), implying that these polymorphisms are highly associated with the low virulence of CVI988. However, RB-1B-based rMDV encoding the wild-type L-Meq exhibited comparable virulence with rMDV encoding RB-1B-Meq, presumably due to the insertion in the transactivation domain (50). Therefore, additional viral factors appear to contribute to its low virulence. Future elucidation of the factors that determine the low virulence of CVI988 could contribute to the development of more effective vaccines against MD.

## MATERIALS AND METHODS

### Cells

CEFs were obtained from 10-day-old fertilized eggs (Iwamura Hatchery Co. Ltd., Shibata, Japan) as described previously (51). CEFs were cultured in Eagle’s minimal essential medium (Nissui Pharmaceutical Co., Ltd., Tokyo, Japan) supplemented with 10% tryptose phosphate broth (Difco Laboratories, Detroit, USA), 0.03% L-glutamine, 100 U/mL penicillin, 100 μg/mL streptomycin, 10% calf serum (Sigma-Aldrich, St. Louis, MO, USA), and 0.1% NaHCO_3_. DF-1 cells, a chicken fibroblast cell line, were cultured in Dulbecco’s modified Eagle’s medium (FUJIFILM Wako Pure Chemical Corporation, Osaka, Japan), containing 0.03% L-glutamine, 100 U/mL penicillin, 100 μg/mL streptomycin, and 10% fetal bovine serum (MP biomedicals, Santa Ana, CA, USA) and incubated at 39°C under 5% CO_2_.

### Plasmids

The expression plasmids for L-Meq were constructed, and mutations were introduced by site-directed mutagenesis, as reported previously (33). The open reading frame (ORF) of L-*Meq* derived from CVI988 (accession number: AF493555) was amplified and cloned into the pCI-neo vector (Promega, Madison, WI, USA). To match the amino acid sequences with those of RB-1B-Meq, we introduced amino acid substitutions at positions 71, 77, and 386 in L-*Meq,* respectively, using the primers listed in Table 4, in addition to the insertion in the transactivation domain, and we designated the protein produced as L-Meq (RB-1B). Next, we constructed an expression plasmid for Meq from RB-1B (accession number: HM488349.1). For the assay to measure transactivation activity, we constructed a c-Jun expression plasmid (38) and reporter plasmids by inserting the promoter regions of pp38, pp14, icp4, gb, chicken cd30, and chicken bcl-2 upstream of the firefly luciferase-coding region in the pGL3-Basic vector (Promega) (38); a pRL-TK *Renilla* luciferase plasmid (Promega) was used as the control plasmid.

**TABLE 4:** Primers used to introduce mutations into the L-*Meq*

Position in Meq	Substitution	Type	Sequence
71	Serine–to–alanine	Forward	5′-GAA TCG TGA CGC CGC TCG GAG AAG ACG-3′
		Reverse	5′-CGT CTT CTC CGA GCG GCG TCA CGA TTC-3′
77	Glutamic-acid–to–lysine	Forward	5′-CGG AGA AGA CGC AGG AAG CAG ACG GAC TAT GTA GAC AAA C -3′
		Reverse	5′-GTT TGT CTA CAT AGT CCG TCT GCT TCC TGC GTC TTC TCC G-3′
326	Threonine-to-isoleucine	Forward	5′-TTCCCTCGGATATTCAGTCTACGGT-3′
		Reverse	5′-ACCGTAGACTGAATATCCGAGGGAA3′
386	Isoleucine–to–threonine	Forward	5′-GTT TCC CTC GGA TAC TCA GTC TAC GGT CT-3′
		Reverse	5′-AGA CCG TAG ACT GAG TAT CCG AGG GAA AC-3′

### Dual-luciferase reporter assay

First, DF-1 cells were seeded in 24-well plates at 2.0 × 10^5^ cells per well and incubated for 24 h. For the reporter assays using icp4, gb, chicken cd30, and chicken bcl-2 promoters, the cells in each well were transfected with 300 ng of Meq/L-Meq expression plasmids, 200 ng of c-Jun expression plasmid, 500 ng of reporter plasmid, and 5 ng of control pRL-TK using Lipofectamine 2000 (Thermo Fisher Scientific, Waltham, MA, USA) according to the manufacturer’s instructions. For the reporter assay using pp38 and pp14 promoters, the cells in each well were transfected with 300 ng of Meq/L-Meq expression plasmids, 500 ng of the reporter plasmid, and 5 ng of control pRL-TK using Lipofectamine 2000 (Thermo Fisher Scientific). The transfected cells were lysed 24 h after transfection using 1 × Passive Lysis Buffer (Promega). Luciferase activity was measured using the Dual-Luciferase Reporter Assay System (Promega) and a Luminescencer-JNR AB-2100 (Atto Corp., Tokyo, Japan). The luminescence intensity of firefly luciferase was normalized to that of *Renilla* luciferase, and the results were reported relative to the value of the luciferase activity in cells transfected with the pCI-neo vector. Three independent experiments were performed in triplicate. The expression levels of each Meq isoform were confirmed by western blotting in each reporter assay.

### Western blotting

To conduct western blotting analysis, antisera against Meq was generated by immunization of a rabbit with a peptide corresponding to the 292–309 amino acid region of Meq (accession number; HM488349.1), which are conserved among most MDV strains (Sigma-Aldrich, MI, USA).

DF-1 cells were seeded in 24-well plates at 2.0 × 10^5^ cells per well and incubated for 24 h. The cells in each well were transfected with 150 ng of Meq/L-Meq expression plasmids using Lipofectamine 2000 (Thermo Fisher Scientific), according to the manufacturer’s instructions. Twenty-four hours after transfection, the cells 100 μg/mL of cycloheximideweres added to each well (Sigma Aldrich) to inhibit protein synthesis. The transfected cells were collected and lysed with Laemmli sample buffers (Bio-Rad, Hercules, CA, USA) containing 5% of 2-mercaptoethanol (Sigma Aldrich) at 0, 3, 6, 9, 12, and 24 h after treatment with cycloheximide. The collected samples were denatured by heating (5 min, 99°C) and electrophoretically separated in 10% SDS-PAGE. The samples were then transferred onto a nitrocellulose membrane (Sigma Aldrich). The membrane was blocked with 3% skimmed milk overnight and incubated with the rabbit anti-Meq antisera (20  µg/mL) and mouse anti-chicken actin antibody (20 μg/mL) as internal control for 1 h at room temperature. The membrane was washed three times with phosphate-buffered saline with Tween 20 (PBS-T) and incubated with anti-rabbit IgG secondary antibody conjugated with HRP (Promega) or anti-mouse IgG_1_ secondary antibody conjugated with HRP (Thermo Fisher Scientific) for 30  min at room temperature, respectively. After washing with PBS-T, the membrane was developed using an immobilized western chemiluminescent HRP substance (EMD Millipore Co., Burlington, MA, USA). Three independent experiments were performed in triplicate.

### Generation of recombinant viruses

We generated RB-1B-based rMDVs encoding Meq or L-Meq (RB-1B), as previously described (33, 50). We first deleted the IRL (pRB-1B_ΔIRL) from the RB-1B genome cloned as a BAC plasmid (52). As the IRL region is rapidly restored during viral reconstitution (17), we replaced *Meq* in the TRL with RB-1B-*Meq* or L-*Meq* (RB-1B) via a two-step Red-mediated mutagenesis as previously described (53, 54). Restriction fragment length polymorphism analysis for the resulting BAC was carried out (33). To screen for clones in which each *Meq*-isoform was accurately inserted, the obtained plasmids encoding each rMDV genome were digested with BamHI-HF (New England Biolabs Japan Inc., Tokyo, Japan) overnight and subjected to electrophoresis on a 0.8% agarose gel as previously described (33). The insertion of each Meq-isoform was further confirmed by PCR and DNA sequencing (40). The BAC was transfected into CEFs using a CalPhos Mammalian Transfection Kit (Takara Bio Inc., Kyoto, Japan) according to the manufacturer’s instructions. The pCAGGS-Cre plasmid (Gene Bridges GmbH, Heidelberg, Germany) was co-transfected to remove the BAC sequence from the virus genome. The reconstituted rMDVs were referred to as vRB-1B_Meq and vRB-1B_L-Meq, respectively, and all viruses were passaged on CEFs and stored in Cell Banker 1 (Nippon Zenyaku Kogyo Co., Ltd., Fukushima, Japan) at −80°C. IRL restoration and BAC sequence deletion in each virus were confirmed by PCR (40). Each virus was titrated using a plaque assay and stored as described previously (55, 56). To confirm if the unexpected mutations in the genome of reconstituted viruses were present, the whole genome of each rMDV in the DNA samples from infected CEFs was determined. Whole genome sequencing was conducted by Hokkaido System Science Co., Ltd. (Hokkaido, Japan) as previously described (57), and we confirmed that no mutations were found in the ORFs of genes previously reported to be associated with MDV virulence, except for *meq* (BioSample accessions: LC849254, LC849255).

### Experimental infection

Fertilized eggs (Iwamura Hatchery Co. Ltd.) from white leghorn chickens were hatched in the laboratory, and the chicks were raised in isolators. The PCR-based detection confirmed that several infectious pathogens, including chicken infectious anemia virus and infectious bursal disease virus, were negative in this study. A total of 83 1-day-old chicks were randomly divided into three groups and housed separately. The chickens were intraperitoneally inoculated with 5,000 PFU of vRB-1B_Meq (*n* = 29), vRB-1B_L-Meq (*n* = 34), or phosphate-buffered saline (PBS, pH 7.4) (*n* = 20) as a negative control. Four chickens per group were euthanized at 7, 14, 28, 35, and 49 dpi, and their blood, spleen, thymus, and tumor lesions were collected. After inoculating the recombinant viruses, we monitored the clinical signs of MD daily for 8 weeks; the infected chickens showing clinical signs during the experimental period were euthanized by collecting heparinized whole blood from the hearts under deep general anesthesia with isoflurane inhalation (Zoetis Japan, Tokyo, Japan), and gross tumor lesions were examined. We also euthanized all remaining chickens, which exhibited no clinical signs on the final day of this experimental infection (56 dpi) (control group; *n* = 4, vRB-1B_Meq-infected group; n = 7), and examined the tumor incidence in the infected chickens. DNA was extracted from whole blood, and the viral loads of each sample were analyzed. Spleens and tumor tissues were dissected with scissors, homogenized, and strained using 40-μm cell strainers (BD Biosciences, San Jose, CA, USA) to obtain cell suspensions, which were washed twice with PBS. Mononuclear cells were isolated from whole blood and spleen cell suspensions by density gradient centrifugation using Percoll solution (GE Healthcare, Chicago, IL, USA) and washed twice with PBS. These separated cells were stored in CELLBANKER 1 (Nippon Zenyaku Kogyo Co., Ltd.) for flow cytometric analysis.

### DNA extraction and qPCR

Total cellular DNA was extracted from the whole blood samples of infected chickens using a DNeasy blood and tissue kit (Qiagen, Tokyo, Japan) according to the manufacturer’s instructions. The viral loads in chickens infected with the recombinant viruses were determined by qPCR using primers specific to the *icp4* gene of MDV. qPCR was performed using TB Green Premix DimerEraser (Takara Bio Inc.) and LightCycler 480 System II (Roche Diagnostics, Mannheim, Germany). The chicken-inducible nitric oxide synthase (*i-nos*) gene was amplified as a reference gene. The viral loads were indicated as ratios of each target gene and *i-nos*. The primers used for the qPCR analyses are listed in Table 5.

**TABLE 5:** Primers used for quantitative polymerase chain reaction

Gene	Type	Sequence
*ICP4*	Forward	5′- GCATCGACAAGCACTTACGG -3′
	Reverse	5′- CGAGAGCGTCGTATTGTTTGG -3′
*iNOS*	Forward	5′- GAGTGGTTTAAGGAGTTGGATCTGA -3′
	Reverse	5′- TTCCAGACCTCCCACCTCAA -3′
*meq*	Forward	5′-GTCCCCCCTCGATCTTTCTC -3′
	Reverse	5′-CGTCTGCTTCCTGCGTCTTC -3′
*COX1*	Forward	5′-ATACTACTTACCGACCGCAACC -3′
	Reverse	5′-AGGATGTAAACTTCGGGGTGAC -3′
*COX2*	Forward	5′-AGGACGGGCTATTATGGGGA -3′
	Reverse	5′-GTGATCTCGACGTCAACACG -3′
*β-actin*	Forward	5′-GAGAAATTGTGCGTGACATCA -3′
	Reverse	5′-CCTGAACCTCTCATTGCCA -3′

### Flow cytometric analysis

PBMCs or cells (5 × 10^5^) isolated from spleen, thymus, or tumor tissues were seeded in 96-well round-bottom plates and washed twice with FACS buffer (PBS supplemented with 1% of bovine serum albumin (Sigma Aldrich)). The cells were blocked with PBS containing 10% chicken serum (Thermo Fisher Scientific) at 25°C for 15 min. Subsequently, the cells were stained with mouse anti-chicken CD3ε mAbs (Southern Biotech, Birmingham, AL, USA) conjugated with PerCP-Cyanine5.5 dye, mouse anti-chicken CD4 mAbs conjugated with PE-Cyanine 7 dye (PE-Cy7; Southern Biotech), mouse anti-chicken CD8β mAbs conjugated with fluorescein-5-isothiocyanate (FITC; Southern Biotech), mouse anti-chicken TCRγδ mAbs conjugated with phycoerythrin (PE; Southern Biotech), and mouse anti-TGF-beta1,2,3 mAbs conjugated with allophycocyanin (APC; R&D systems, Abingdon, UK), or mouse IgG isotype control (R&D systems) for 30 min at 4°C in the dark. The gating strategy is shown in Fig. 4. In addition, we further analyzed γδ, CD4, and CD8 T cell subsets. The cells were stained with mouse anti-chicken CD3ε mAbs conjugated with PerCP-Cyanine5.5 dye, mouse anti-chicken CD4 mAbs conjugated with PE-Cy7, mouse anti-chicken CD8β mAbs conjugated with FITC, mouse anti-chicken CD4 mAbs conjugated with PE, and mouse anti-chicken CD8α mAbs conjugated with APC (Southern Biotech) for 30 min at 4°C in the dark. The gating strategy is shown in Fig. 6. Dead cells were stained using Fixable Viability Dye eFluor780 (Thermo Fisher Scientific). Then, the cells were washed twice with 200 µL of FACS buffer and analyzed using a FACSLyric flow cytometer (BD Biosciences).

### RNA sequencing

Total RNA was extracted from tumor lesions of two chickens from each rMDV-infected group using an RNeasy Plus Mini Kit (Qiagen) according to the manufacturer’s instructions. RNA-seq was conducted by Hokkaido System Science Co., Ltd. (Hokkaido, Japan).

The qualities of the total RNA samples were assessed using an Agilent 2100 Bioanalyzer (Agilent Technologies, Böblingen, Germany). Subsequently, a cDNA library was constructed using a NEBNext Ultra Directional RNA Library Prep Kit (New England Biolabs Japan Inc.), according to the standard protocol. Paired-end sequencing was then conducted on the Illumina NovaSeq (Illumina, Inc., San Diego, CA, USA) following the manufacturer’s instructions. The adaptor sequences that were added to construct the cDNA library were trimmed from raw reads using cutadapt v1.18 (https://cutadapt.readthedocs.org/en/stable). The regions with QVs lower than 20 were further trimmed, and the read sequences with more than 75 bp were used for further analyses. The raw sequence data were deposited in DDBJ/ENA/GeneBank (BioSample accessions: SAMD00731360, SAMD00731361, SAMD00731362, SAMD00731363). To analyze the expression intensities, the trimmed reads were mapped to the transcript sequences of *Gallus* GRCg6a (Release 105) from the Ensemble Database as the reference sequence, and TPM was calculated using Salmon v1.2.1 (https://github.com/COMBINE-lab/salmon). In this process, the chicken genome sequence from the same database was used as a decoy sequence to improve the mapping accuracy. The TPM values were used for correlation analysis among samples. The differences in expression intensities between each group were analyzed via GO analysis by DAVID Bioinformatics Resources (https://davidbioinformatics.nih.gov/summary.jsp) using the trimmed reads.

### Statistical analyses

Statistical analyses were performed using R Statistical Software (version 4.0.3; R Foundation for Statistical Computing, Vienna, Austria). The multi-step growth kinetics were analyzed using the Mann–Whitney U test. T cell dynamics up to 35 dpi and the percentage of T cells in the chickens that developed lymphomas was analyzed using the Kruskal–Wallis test and Dunn’s test. T cell dynamics after 49 dpi were analyzed using the Mann–Whitney U test. Tumor incidence was analyzed using Fisher’s exact test. The transactivation activities were analyzed using Tukey’s multiple comparison test. The TPM values for each sample were analyzed using the Spearman rank correlation test. Statistical significance was set at *P* < 0.05.

## Data Availability

The data sets used and analyzed in this study are available from the corresponding author on reasonable request.

## SUPPLEMENTAL MATERIAL

Fig. S1 spectrum.03368-24-s0001.tiff Uncropped images for Fig. 1H.

ASM does not own the copyrights to Supplemental Material that may be linked to, or accessed through, an article. The authors have granted ASM a non-exclusive, world-wide license to publish the Supplemental Material files. Please contact the corresponding author directly for reuse.
