# Supplementary figures and images for: Amino acid insertion in the Meq protein of Marek’s disease virus, an avian oncogenic herpesvirus, accelerates tumorigenesis
Source: Microbiol Spectr. 2025 Jul 17;13(8):e03368-24. doi: 10.1128/spectrum.03368-24 (PMC12323629; doi:10.1128/spectrum.03368-24)

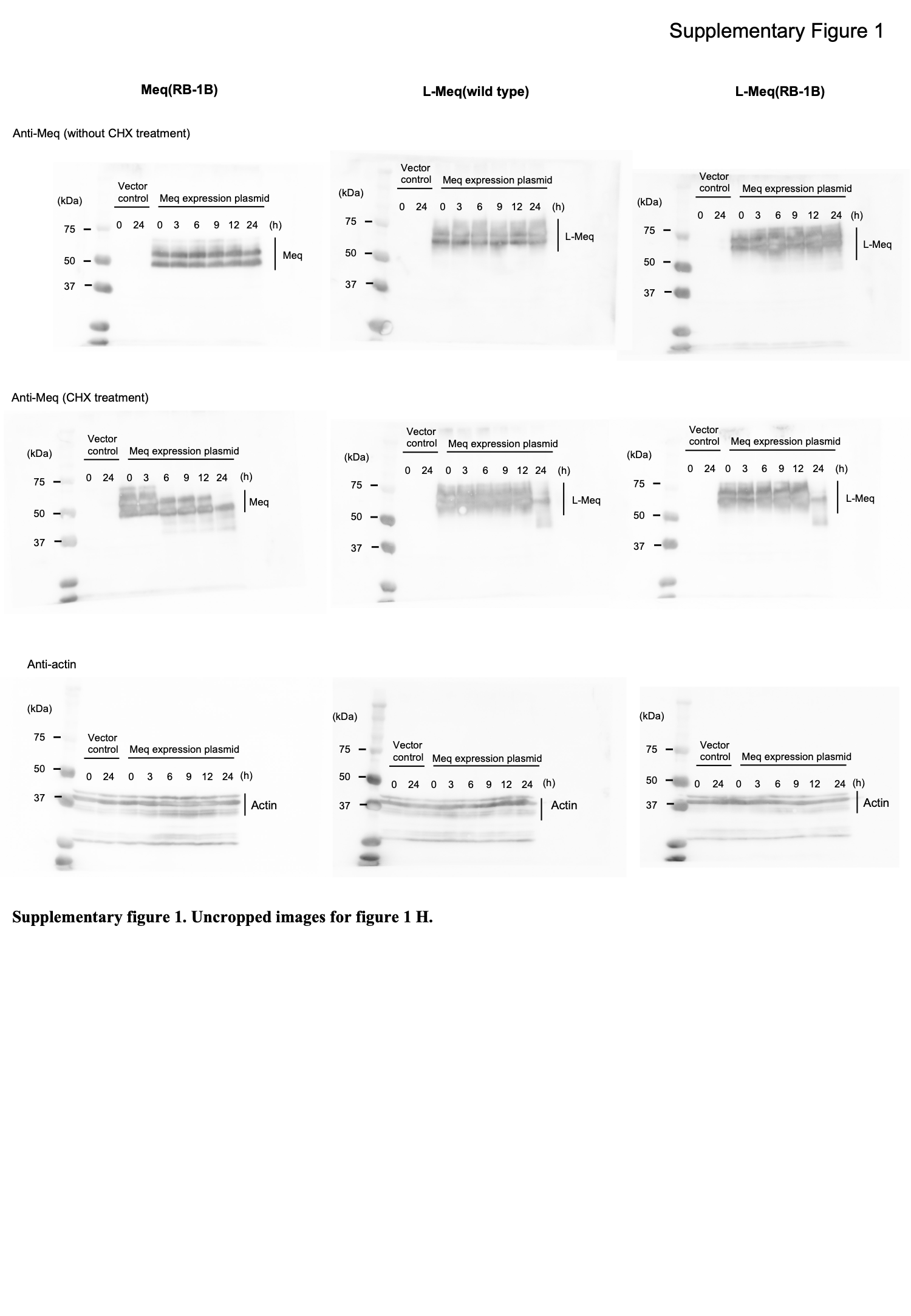

Supplement: Fig. S1 — Uncropped images for Fig. 1H. [file spectrum.03368-24-s0001.tiff]
